# The Effect of Lithium Iodide to the Properties of Carboxymethyl κ-Carrageenan/Carboxymethyl Cellulose Polymer Electrolyte and Dye-Sensitized Solar Cell Performance

**DOI:** 10.3390/polym8050163

**Published:** 2016-05-13

**Authors:** Siti Rudhziah Che Balian, Azizan Ahmad, Nor Sabirin Mohamed

**Affiliations:** 1 Pusat Asasi, Universiti Teknologi MARA, Cawangan Selangor, Kampus Dengkil, 43800 Dengkil, Malaysia; 2 Institut Siswazah, Universiti Malaya, 50603 Kuala Lumpur, Malaysia; 3 Sekolah Sains Kimia dan Teknologi Makanan, Universiti Kebangsaan Malaysia, 43600 Bangi, Malaysia; azizan@ukm.edu.my; 4 Pusat Asasi Sains, Universiti Malaya, 50603 Kuala Lumpur, Malaysia; nsabirin@um.edu.my

**Keywords:** κ-carrageenan, kenaf-cellulose, polymer blend, polymer electrolytes, dye sensitized solar cell

## Abstract

This study was undertaken to investigate the solid biopolymer electrolytes based on a carboxymethyl κ-carrageenan/carboxymethyl cellulose blend complexed with lithium iodide of various weight ratios. The complexation of the doping salt with the polymer blend was confirmed by Fourier transform infrared spectroscopy. Ionic conductivity of the film was determined by impedance spectroscopy in the frequency range of 10 Hz to 4 MHz and in the temperature range of 303–338 K. The ionic conductivity increased with the increase in lithium iodide concentration as well as temperature. The membrane comprising 30 wt % of lithium iodide was found to give the highest conductivity of 3.89 × 10^−3^ S·cm^−1^ at room temperature. The increase in conductivity was associated with the increase in the number as well as the mobility of the charge carries. The conductivity increase with temperature followed the Vogel–Tamman–Fulcher model. The fabricated dye-sensitive solar cell, FTO/TiO_2_-dye/CMKC/CMCE-LiI (30 wt %) +I_2_/Pt exhibited the highest conversion efficiency of 0.11% at a light intensity of 100 mW·cm^−2^. This indicated that the biopolymer blend electrolyte system has potential for use in dye-sensitized solar cells.

## 1. Introduction

In 1991, Grätzel and co-workers developed the most well-known unconventional photovoltaic system, which is the dye-sensitized nanostructured solar cell (DSSC), using organic liquid electrolytes [1,2]. This type of solar cell has attracted a great deal of attention due to its low production cost, simple structure and high power conversion efficiency. However, the most significant drawback of DSSCs using liquid electrolytes is the poor long-term stability due to the volatility of the electrolyte that contains organic solvent. This problem can be overcome by replacing the liquid electrolyte with a gel electrolyte or solid polymer electrolyte. The gel electrolyte shows better conductivity as well as high efficiency although the liquid encapsulated inside the gel pores creates a problem during solar cell sealing and hence affects the long-term stability of the cells [2,3].

The solid polymer electrolyte seems to be a novel alternative electrolyte in DSSCs due to it being completely solid in nature, with excellent properties such as a good conduction, low cost and good stability; furthermore, it is easy to fabricate. They have obtained growing interest from the point of practical applications. Lee and co-workers (2008) studied poly(ethylene oxide)/poly(dimethylsiloxane) (PEO/PDMS) blend solid polymer electrolyte and its dye-sensitized solar cell applications. Their DSSC employing the PEO/PDMS blend complexed with LiI showed energy conversion efficiencies of 0.48% and 1.35% at 100 and 10 mW·cm^−2^, respectively [4]. Meanwhile, Roh *et al.* (2010) reported that DSSCs fabricated using the PVC-*g*-POEM/(LiI + MPII)/PEG electrolyte exhibited an energy conversion efficiency of ~5.0% at 100 mW·cm^−2^ [5]. Even though many studies on DSSCs employing polymer electrolytes have been done, most of the studies reported in the literature used petrochemical-based polymers, which are associated with environmental issues. In order to reduce the dependence on petrochemical-based polymers of electrolytes, bio-based polymers may be applied as hosts [6,7,8]. Su’ait *et al.* (2014) developed a solid-state DSSC cell from bio-based polyurethane (PU). They reported that their solid-state DSSC with the configuration of FTO/TiO_2_-dye/PU-LiI-I_2_/Pt showed a photovoltaic response with a *J*sc of 0.06 mA·cm^−2^, an open circuit voltage *V*oc of 0.14 V, a fill factor *ff* of 0.26 and an energy conversion efficiency *η* of 0.003% [7].

In an effort to reduce dependency on petrochemical-based electrolytes, this study focused on developing electrolytes based on a biopolymer blend by using modified natural polymers, namely carboxymethyl κ-carrageenan (CMKC) and carboxymethyl cellulose (CMCE), as the host polymers for potential use in DSSCs. In our previous study, we succeeded in preparing a polymer blend based on CMKC/CMCE. The free-standing blend film of CMKC/CMCE with a weight ratio of 60:40 was the most flexible, exhibited a conductivity of 3.25 × 10^−4^ S·cm^−1^ and had the lowest glass transition of −13.5 °C [9]. We also prepared biopolymer electrolytes based on the CMKC/CMCE blend doped with ammonium iodide. The result showed that the system containing 30 wt % of NH_4_I exhibited the highest room conductivity of 2.41 × 10^−3^ S·cm^−1^ and a glass transition temperature of −5.7 °C. The DSSC fabricated using this system exhibited an energy conversion efficiency of 0.13% [10]. Although biopolymers doped with ammonium salt are well known, biopolymers doped with lithium salts have been very rarely studied for use as electrolytes of DSCCs. Therefore, in the present work, CMKC/CMCE doped with LiI salt was prepared and the effect of the salt on the properties of the CMKC/CMCE blend film was investigated. We have chosen lithium salt because the size of its cation is the smallest compared to other types of salts. It is generally believed that the conductivity of the electrolytes with smaller cations should be higher, possibly due to the higher mobility of smaller cations [11].

## 2. Materials and Methods

### 2.1. Materials

**Figure 1:**
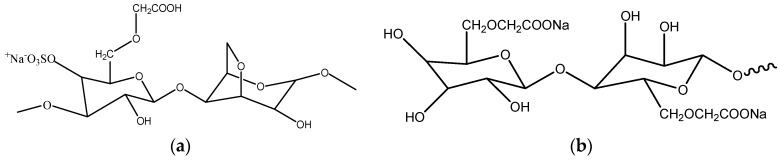
(**a**) Structure of CMKC; (**b**) Structure of CMCE.

κ-carrageenan was supplied by Takarra Sdn. Bhd., Sabah, Malaysia. Meanwhile, Kenaf fibers were obtained from KFI Sdn. Bhd., Kelantan, Malaysia. Sulfuric acid (98%), sodium hydroxide (99%), sodium chlorite (80%), glacial acetid acid (99.5%), distilled water (1–0.1 µS·cm^−1^), isopropanol, monochloroacetic acid and LiI, iodine (I_2_), di-tetrabutylammonium cis-bis(isothiocynato) bis(2,2′-bipyridyl-4,4′-dicarboxylato ruthenium (II) (N719) and platinum (Pt) were purchased from from SYSTERM-chemAR and Sigma-Aldrich (St. Louis, MO, USA). Meanwhile, the TiO_2_ paste DSL 18 NR-AO was supplied from Dyesol (New South Wales, Australia). All materials were used as received. The cellulose was extracted from kenaf fiber. Both materials were synthesized according to the method proposed by Sun *et al.* [12] to obtain CMKC and CMCE. The structures of the polymers are shown in Figure 1. The details of the synthesis procedures were given in Rudhziah *et al.* [9]. The degree of substitution (DS) of the carboxymethyl for each sample was estimated using potentiometric titration [13]. The DS of CMKC and CMCE obtained in this study are 0.85 and 1.93.

### 2.2. Preparation and Characterization of Electrolytes

Electrolytes were prepared using solution casting technique. First 0.6 g of CMKC and 0.4 g of CMCE were dissolved in 50 mL of 1% (*v*/*v*) of aqueous acetic acid solution. The solution was stirred at 40 °C for a few hours. Then 10% of LiI was then added to the solution. The weight percentage of the salt was calculated using the equation:

(1) $$B\%=\frac{B}{A}\times 100\;\%$$

where *A* and *B* are the weight of CMKC/CMC and the weight of salt, respectively. The solution was further stirred at room temperature for a few hours and cast into Petri dishes and allowed to evaporate slowly at ambient temperature. The procedures were repeated to prepare other samples. The obtained free-standing films with thickness between 0.20 and 0.30 mm were then kept in a desiccator for further drying.

In order to study the interactions of the polymer host and the incorporated salt, FTIR spectroscopy was performed using Perkin Elmer Frontier spectrophotometer (Waltham, MA, USA). The spectrophotometer was equipped with an Attenuated Total Reflection accessory with a germanium crystal. The sample was put on the germanium crystal and infrared light was passed through the sample in the wavenumber range from 4000 to 550 cm^−1^ at a resolution of 1 cm^−1^. The glass transition temperature, *T*_g_ of the CMKC/CMCE blend–based electrolyte films were determined using Perkin Elmer DMA 8000 in tension mode. Rectangular strip specimens (width 10 mm, length 20 mm, thickness 0.20 to 0.30 mm) were used. The dual cantilever mode of deformation was used at the test temperatures ranging from −40 to 100 °C with a heating rate of 2 °C·min^−1^ at the frequency of 1 Hz. The *T*_g_ values of the films were determined from the peak of the tan δ-*T* curves. The characterization of electrical properties of the electrolyte films were carried out using a high frequency response analyzer (Solartron 1260, Hampshire, UK) in the frequency range of 10 Hz to 4 MHz with 10 mV voltage amplitude at various temperatures ranging from 300 to 333 K. In order to clarify the conduction mechanism, measured impedance have been converted in terms of the real component of permittivity, ε_r_. The value ε_r_ was calculated using the equation:

(2) $$\varepsilon_{r}=\frac{Z_{i}}{\omega C_{o}({Z_{r}}^{2}+{Z_{i}}^{2})}$$

where *C*_o_ is capacity of the empty cell. The sample for the measurement was sandwiched between stainless steel blocking electrodes with a contact surface area of 3.142 cm^2^.

### 2.3. Preparation and Characterization of Solid-State DSSC

Dye-sensitized solar cells with active area of about 1 cm^2^ were fabricated using the electrolyte containing 10–30 wt % of LiI. For the preparation of the DSSC photoelectrode, the TiO_2_ paste was spread on FTO conducting glass using doctor blade technique followed by sintering process at 450 °C for 30 min. This electrode was then immersed into the solution of the dye N719 for 24 h. Meanwhile, the platinum FTO glass counter-electrode was prepared by brush painting technique and heating at 450 °C for 30 min. For redox couple formation in the DSSC, iodine was added into the electrolyte. The (CMKC/CMCE+LiI):I_2_ ratio was (10:1). The electrolytes was then cast onto TiO_2_/dye photoelectrode and heated at 50 °C to form film. Finally, the electrolyte was sandwiched between the TiO_2_/dye photoelectrode and platinum counter-electrode. The performance of the fabricated DSSCs was examined under white light illumination (100 mW·cm^−2^) using the Wonatech Zive MP2 multichannel electrochemical workstation. The fill factor (*ff*) was determined using the following equation:

(3) $$ff=\frac{V_{\text{max}}\times J_{\max}}{V_{\text{oc}}\times J_{\text{sc}}}$$

where *J*_sc_ is the short-circuit current density (mA·cm^−2^), *V*_oc_ is the open-circuit voltage (V), and *J*_max_ (mA·cm^−2^) and *V*_max_ (V) are the current density and voltage in the *J*–*V* curves, respectively, at the maximum power output. The external light to energy conversion efficiency *η* was obtained from the following equation:

(4) $$\eta (\%)=\frac{V_{\text{oc}}\cdot J_{\text{sc}}\cdot ff}{P_{\text{in}}}\times 100\%$$

where *P*_in_ is the incident light power.

## 3. Results

### 3.1. FTIR Study

**Figure 2:**
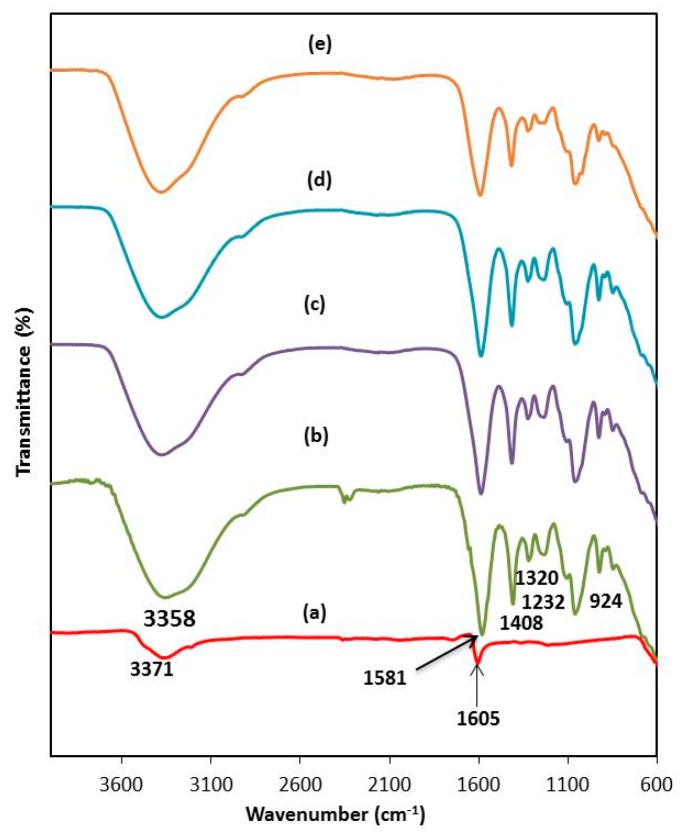
FTIR spectra of (**a**) LiI and CMKC/CMCE-salt films containing (**b**) 0; (**c**) 10; (**d**) 20 and (**e**) 30 wt % LiI.

In this study, the FTIR spectra of CMKC/CMCE-LiI with various concentrations of LiI were taken and analyzed in order to study the formation of polymer host-salt complexes. The recorded FTIR spectra are presented in Figure 2 and Table 1. The bands at 3358, 1581, 1408, 1320, 1232 and 924 cm^−1^ correspond to OH stretching, COO^−^ asymmetrical stretching of carboxylate anion, COO^−^ symmetric stretching, −CH_2_ scissoring, O=S=O symmetric vibration and C−O−C stretching of CMKC/CMCE (0 wt % Li) [14,15]. With the addition of lithium salt, the band at 3358 cm^−1^ which belongs to OH functional groups shifted to the higher wavenumber of 3377 cm^−1^ due to the hygroscopic nature of the lithium salt. Besides this, the bands of functional groups of COO^−^ asymmetrical of the carboxylate anion and COO^−^ symmetric stretching of CMKC/CMCE also shifted to higher wavenumbers. This is due to electrostatic interactions between the C−O− in the acetate ions (acetic acid) and the carboxymethyl ions being stronger compared to the ion dipole interactions that form between the oxygen atom in C=O with the lithium ion. These bands are also observed to be reduced in intensity. These shiftings indicate the existence of an interaction between lithium ions from doping salt and oxygen atoms of the copolymer host [7].

### 3.2. DMA Study

**Figure 3:**
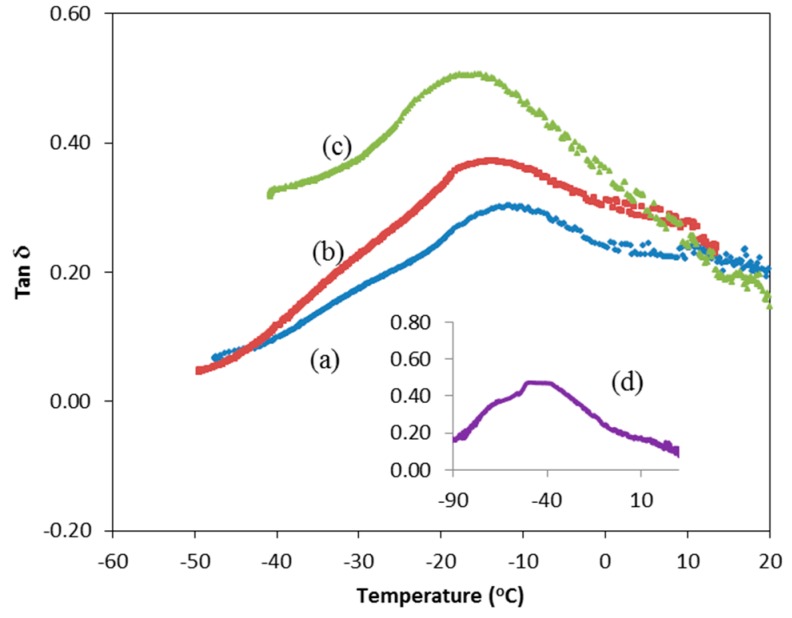
Tan δ *versus* temperature for CMKC/CMCE films with (**a**) 0; (**b**) 10; (**c**) 20 and (**d**) 30 wt % LiI.

Figure 3 displays the tan δ-temperature curves while Table 2 presents the *T*_g_ variation for CMKC/CMCE-LiI salt complexes as a function of the lithium salt concentration. The damping curves presented in Figure 3 show the main relaxation process in the amorphous region of the CMKC/CMCE-LiI. The damping peak corresponds to the *T*_g_ of CMKC/CMKC (0 wt % LiI) that is observed at −13.5 °C. The value of *T*_g_ decreases gradually with the incorporation of salt (10, 20 and 30 wt %). The decrease of the *T*_g_ value with the addition of salt could be due to the plasticizing effect of the salt which results in a weakening of the dipole-dipole interactions between the polymer chains. This plasticizing effect softens the polymer backbone and thus produces a flexible polymer backbone. The flexible polymer backbone increases the segmental motion of the polymer matrix, hence producing free volume. The existence of the free volume enables ions to migrate easily [16].

### 3.3. Room Temperature Conductivity Study

Table 3 lists the average room temperature conductivity of the polymer electrolyte membranes investigated in this study. The conductivity values are observed to increase gradually with the salt concentration from 10 to 30 wt %. The highest ionic conductivity of 3.89 × 10^−3^ S·cm^−1^ is observed at a salt concentration of 30 wt %. The increase in conductivity with the increasing salt concentration can be attributed to the increase in the number of charge carriers. The conductivity of electrolytes is given as:

(5) σ=n⋅q⋅μ

where *n* is the density of the mobile charge carriers, *q* is the charge of the ions and µ is the mobility of the charge carriers. Therefore, any increase in either *n* or µ leads to an increase in the conductivity.

**Figure 4:**
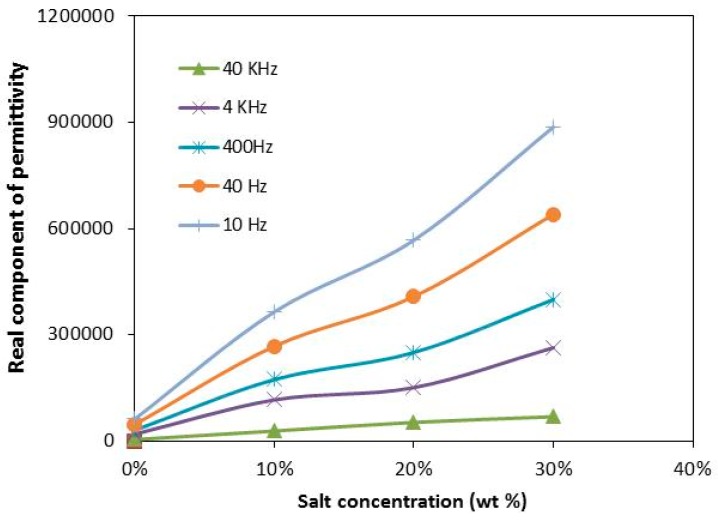
The plots of the real component of permittivity variation concentration at various frequencies for CMKC/CMCE-LiI films.

The plots of the real component of permittivity variation with salt concentration for selected frequencies from 10 Hz to 40 kHz at room temperature are shown in Figure 4. High values of the real component of permittivity are observed at low frequency. At low frequency, the period of the applied electric field is long and the ions accumulate at the electrode/electrolyte interface. The addition of LiI salt also increases the real component of permittivity. The increase in the real component of permittivity with the salt concentration gives a reflection of an increase in the number of charge carriers [17].

### 3.4. Conductivity at Various Temperatures

**Figure 5:**
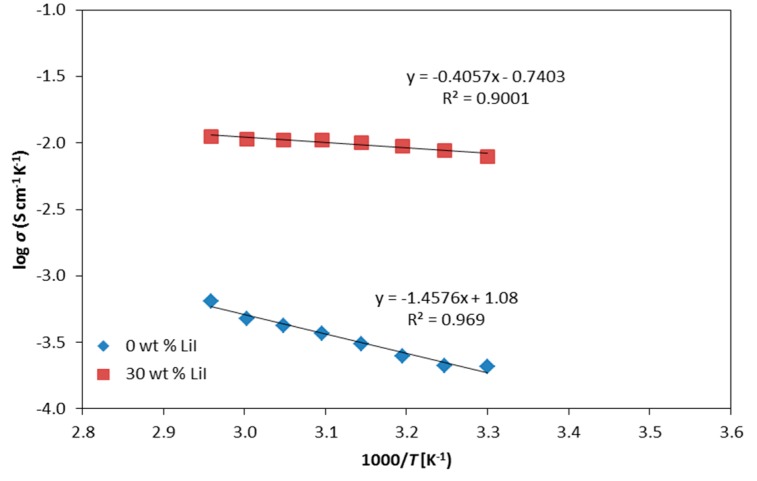
Arrhenius plots for CMKC/CMCE-LiI films.

Figure 5 demonstrates the temperature-dependent conductivity of CMKC/CMCE-LiI films. The conductivity of the system is observed to increase with the reciprocal value of the temperature. The non-linear σ *vs.* 1000/T plots suggests that ion transport in the polymer electrolytes exhibits the Vogel–Tammann–Fulcher (VTF) theory which is associated with the free volume model.

**Figure 6:**
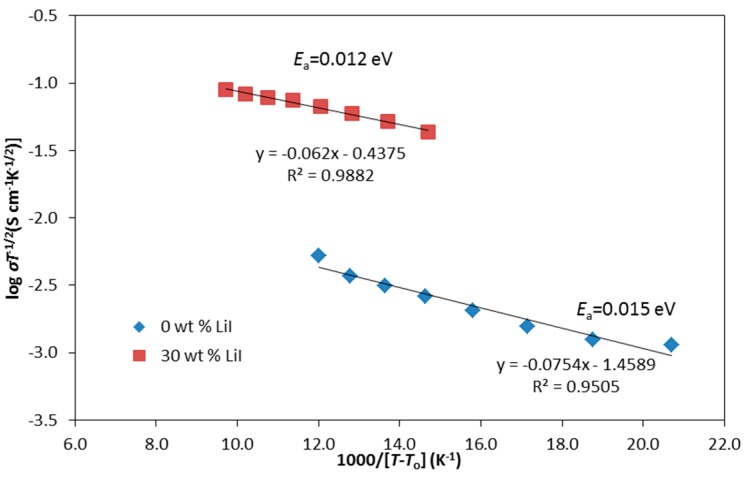
VTF plots for CMKC/CMCE-LiI films.

The temperature-dependent conductivity of the biopolymer electrolytes follows the VTF model which is expressed as follows:

(6) σ=σoT−12exp (−EaκB(T−To))

where σ_o_ is the pre-exponential factor which is proportional to the number of charge carriers, *E*_a_ is the activation energy for conduction which is related to polymer segmental motion, $\kappa_{B}$ is the Boltzmann constant, *T* is the absolute temperature and *T*_o_ is the ideal vitreous transition temperature at which the polymer segments start to move. *T*_0_ is also the reference temperature taken as the glass transition temperature, *T*_g_. On the other hand, experimental *T*_g_ is determined from DMA analysis. Figure 6 shows all the plots are well fitted with the VTF rule. The increase of ionic conductivity with temperature is interpreted as being due to a hopping mechanism between coordination sites, local structural relaxations and the segmental motion of polymers. As the free volume progressively increases with temperature, the polymer chain acquires faster internal modes in which bond rotation produces segmental motion. This, in turn, favors the hopping of inter-chain and intra-chain ion movements and the conductivity of the polymer electrolyte becomes high [18]. Figure 4 also shows that *E*_a_ decreases with the addition of LiI salt. This observation shows that CMKC/CMCE-LiI exhibits higher flexibility of the polymer backbone coupled with an increased segmental mobility of its chains. Therefore, it requires low activation energy for the hopping process.

### 3.5. J–V Performance

**Figure 7:**
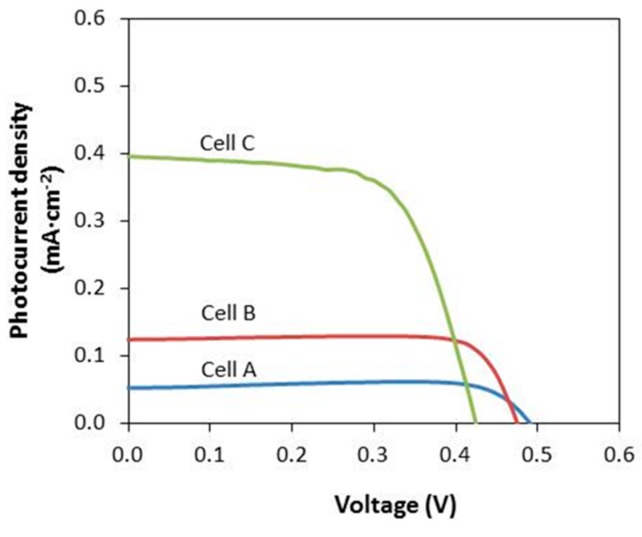
Photocurrent density-voltage for DSSC.

Figure 7 presents the photocurrent density–voltage curves of CMKC/CMCE-LiI-based solid-state DSSC at a light intensity of 100 mW·cm^−1^. Meanwhile, the values of *V*_OC,_ *J*_SC,_ *ff* and η% are tabulated in Table 4. The energy conversion efficiency η% of Cell A is 0.02% with a *V*_OC_ of 0.498 V, *J*_SC_ of 0.05 mA·cm^−2^ and *ff* of 0.90, while the energy conversion efficiency η% of Cell B is 0.05% with a *V*_OC_ of 0.492 V, *J*_SC_ of 0.12 mA·cm^−2^ and *ff* of 0.80. The energy conversion efficiency η% of Cell C is 0.11% with a *V*_OC_ of 0.492 V, *J*_SC_ of 0.42 mA·cm^−2^ and *ff* of 0.57. It is observed that Cell A shows a lower *J*_SC_ and higher *V*_OC_ than the DSSC of Cell B and Cell C. The lower value of *J*_SC_ of Cell A is ascribed to its lower ionic conductivity. A higher resistance to ion migration reduces the supply of I_3_^−^ to the Pt counter-electrode. This causes a depletion of I_3_^−^ and also retards the kinetics of dye regeneration and therefore decreases the *J*_SC_ value. Meanwhile, the slight increase of *V*_OC_ for the DSSC (Cell A) is related to the reduction of the back electron-transfer reaction [19]. The values of *J*_SC_ and the solar cell efficiency increase with the addition of LiI salt. This indicates that the *J*sc value is directly related to the conductivity of the CMKC/CMCE-LiI electrolyte. The energy conversion efficiency DSSC fabricated using CMKC/CMCE–(30 wt %) LiI is found lower than the previously reported DSSC fabricated using CMKC/CMCE–(30 wt %) NH_4_I, although the conductivity of the film in this study is slightly higher. The lower energy efficiency of the DSSC fabricated in this study may be due to the formation of Ti^3+^ species which trap electrons in localized states upon the intercalation of Li^+^ ions into a TiO_2_ nanostructured photoelectrode [20]. Even though solid-state DSSCs employing CMKC/CMCE electrolytes showed a response under the light intensity of 100 mW·cm^−2^ and exhibited promising potentials for the DSSC, the performance of the DSSC in this study is low. Therefore, further work should be carried out to enhance the DSSC performance. This can be done using mixed cation salts such as the mix of LiI and KI or LiI and tetrahexylammonium iodide as a doping salt in the CMKC/CMCE blend system. It is believed that an electrolyte system containing mixed cations gives a better solar cell performance compared to a single cation system due to the beneficial effects from both types of cations.

## 4. Conclusions

Electrolytes based on the blend of modified natural polymers CMKC and CMCE were successfully developed for potential use in solid-state DSSCs. The glass transition, dielectric constant and conductivity of the CMKC/CMCE blend electrolytes were observed to depend on the LiI salt concentration. The increase in conductivity was attributed to the increase in the number and mobility of charge carriers. The CMKC/CMCE blend system containing 30 wt % LiI exhibited the lowest *T*_g_ of −43.0 °C and the highest room temperature conductivity of 3.89 × 10^−3^ S·cm^−1^. The values of *J*_sc_ and the solar cell efficiency of the fabricated DSSCs were also observed to depend on the LiI salt. The cell fabricated using the best conducting system, Cell C, exhibited the highest photoelectric conversion efficiency η% of 0.11%.

## Figures and Tables

**Table 1:** FTIR band assignments and wavenumbers of CMKC/CMCE-based polymer electrolyte.

Band assignment	Wavenumber (cm^−1^)			
	0 wt % LiI	10 wt % LiI	20 wt % LiI	30 wt % LiI
OH stretching	3,358	3,377	3,378	3,379
COO− asymmetrical of carboxylate anion	1,581	1,587	1,589	1,591
COO– symmetric stretching	1,408	1,414	1,415	1,416
−CH_2_ scissoring	1,320	1,324	1,324	1,324
O=S=O symmetric vibration	1,232	1,242	1,233	1,235
C−O−C stretching	924	927	927	927

**Table 2:** Glass transition temperature for CMKC/CMCE-LiI films.

Concentration of LiI (wt %)	Glass transition temperature, *T*_g_ (°C)
0	−13.5
10	−14.5
20	−16.7
30	−43.0

**Table 3:** Conductivity and relative number of charge carriers for the CMKC/CMCE-LiI films at room temperature.

CMKC/CMCE:Salt	Conductivity, (σ ± Δσ) (S·cm^−1^)
100:0	(3.25 ± 0.25) × 10^−4^
90:10	(1.66 ± 0.16) × 10^−3^
80:20	(2.73 ± 0.09 ) × 10^−3^
70:30	(3.89 ± 0.27) × 10^−3^

**Table 4:** *J*–*V* performance of FTO/TiO_2_/Electrolyte+I_2_/pt/FTO.

CMKC/CMCE:LiI (wt %)	*I_2_* (M)	*V*_oc_ (V)	*J*_sc_ (mA·cm^−2^)	*ff*	η%
10 (Cell A)	0.01	0.498	0.05	0.90	0.02
20 (Cell B)	0.02	0.492	0.12	0.80	0.05
30 (Cell C)	0.03	0.492	0.40	0.57	0.11
